# Simulation of Laser-Heating and Energetic Plasma Plume Expansion in Pulsed Laser Deposition of Y_3_Fe_5_O_12_

**DOI:** 10.3390/mi13112012

**Published:** 2022-11-18

**Authors:** Joko Suwardy, Muzakkiy Putra Muhammad Akhir, Robi Kurniawan, Beni Rio Hermanto, Isa Anshori, Mohammad Hamzah Fauzi

**Affiliations:** 1 Research Center for Quantum Physics, National Research and Innovation Agency (BRIN), South Tangerang 15314, Indonesia; 2 Department of Physics, Faculty of Mathematics and Natural Sciences, Universitas Negeri Malang, Jl. Semarang No. 5, Malang 65145, Indonesia; 3 Biomedical Engineering Department, School of Electrical Engineering and Informatics, Bandung Institute of Technology, Bandung 40132, Indonesia; 4 Research Collaboration Center for Quantum Technology 2.0, Bandung 40132, Indonesia

**Keywords:** pulsed laser deposition, Y_3_Fe_5_O_12_, plasma dynamics

## Abstract

In the present study, numerically iterative models are employed to study two processes involved in the pulsed laser deposition of an Y_3_Fe_5_O_12_ target. The 1D conduction heat model is used to evaluate the temperature of the target irradiated by a nano-second pulse laser, taking into account the plasma shielding effect. Further, the gas dynamics model is employed to simulate the kinetic of plasma plume expansion. The results may be important in obtaining high-quality Y_3_Fe_5_O_12_ thin films.

## 1. Introduction

The ferrimagnetic insulator yttrium iron garnet (Y_3_Fe_5_O_12_), often abbreviated as YIG, has become an attractive material in the field of quantum spintronics. Having a low Gilbert damping value, YIG is widely used to investigate various quantum phenomena, such as spin waves, spin pumping, the inverse spin Hall effect, and spin caloritronic [1,2,3,4]. A high-quality YIG thin film is required to observe these quantum effects.

One of the most powerful techniques to grow complex oxide thin films, such as YIG, is pulsed laser deposition (PLD) [5]. A recent report claimed a successful deposition of YIG thin film on a gallium gadolinium garnet (GGG) substrate using room temperature PLD followed by a post-annealing treatment. This YIG has a monocrystalline structure with a very low damping parameter ($\alpha =6\times 10^{-5}$) [6]. However, the use of garnet substrates such as GGG limits its practical application. Considerable efforts have been put forward to grow YIG on commercially compatible chip substrates, e.g., silicon [7,8,9]. However, its properties still fall behind that of YIG/GGG due to the smaller grain size and cracking of the film [7,8].

Although the procedure of obtaining laser-ablated material is simple, involving a focus pulsed laser with an intensity above the threshold ablating the material target, the physics incorporated therein is overly complex. Various interdependence processes take place such as target-heating, melting, vaporization, ionization, plasma formation, plasma hydrodynamic expansion, shock wave generation, and so on [10,11,12]. In general, based on the interactions, the processes can be divided into three major zones:

1. Interaction between the laser and the target material, which results in evaporation on the target surface [13,14,15,16,17,18],
2. Interaction between the material gas and the laser, which produces plasma [19,20], and
3. Plasma plume expansion [21,22,23].

The first two interactions happen during the laser pulse, while the latter zone occurs when the laser irradiation has stopped.

In this study, we employed numerically iterative models to investigate two crucial processes in the pulsed laser deposition: laser-heating of the target and plasma plume expansion. These two processes govern the kinetic of particles deposited on the substrate. In the calculation, we employed the thermophysical properties of the YIG target. The results are applicable for experimentally grown YIG thin film on commercially compatible chip substrates, e.g., silicon substrate.

## 2. Theoretical Formulation

### 2.1. Laser-Target Interaction

**Figure 1:**
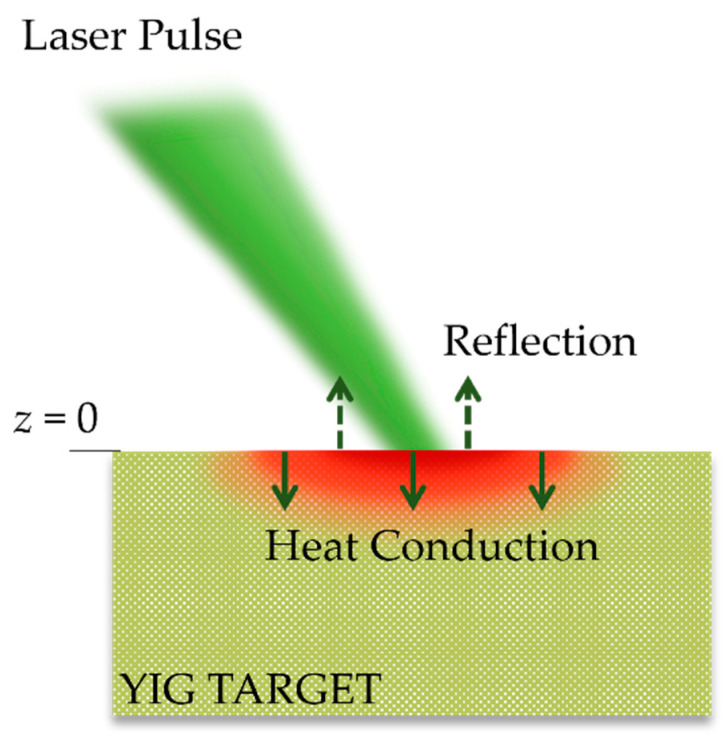
Schematic of laser irradiation of YIG target. The target surface is denoted by *z* = 0. The up and down arrows represent the laser energy that is reflected and absorbed by the target.

Figure 1 illustrates the schematic of the laser heating the target material. A single pulse of nano-second laser was focused onto the YIG target surface. Upon receiving the pulse, the target absorbed some portion of laser energy and reflected the rest. The absorbed laser energy can be expressed as the following source term:

(1) $$\dot{S}=\mu \left(1-R\right)I\left(t\right)e^{-\mu z},$$

where R is the optical reflectivity of YIG and μ=4πk/λ, is the absorption coefficient of YIG. For simplicity, R is assumed to be independent of temperature. Here, λ = 532 nm is the laser wavelength and k is the extinction coefficient. The temporal pulse intensity I(t) is expressed in a Gaussian distribution:

(2) $$I\left(t\right)=\frac{Aexp{\left(\frac{-4\ln\left(2\right){\left(t-t_{c}\right)}^{2}}{w^{2}}\right)}^{}}{w\sqrt{\frac{\pi }{4ln\left(2\right)}}},$$

where w is the full-width half maximum (FWHM) of the laser pulse, A is the area and $t_{c}$ is the center of the Gaussian profile. Here, we used the property of Nd:YAG Continuum laser Surelite III, which is available in our PLD system, with a laser energy of 446 mJ, beam diameter (d) = 9.5 mm, and w is 5 ns. The calculated maximum intensity of $1.26\times 10^{8}$ W/cm^2^ and pulse duration, $t_{L}$ = 15 ns is obtained.

The heat transfer of the nano-second laser-radiated YIG before melting (*t*_m_) can be expressed by a 1D heat conduction equation as follows [13]:

(3) $$C_{s}\rho_{s}\frac{\partial T\left(t,z\right)}{\partial t}=\frac{\partial }{\partial z}\;\left(K_{s}\frac{\partial T\left(t,z\right)}{\partial z}\right)+\dot{S},$$

where $C_{s},\rho_{s},\;K_{s},$ are the density, specific heat, and thermal conductivity of the solid phase YIG, respectively. As shown in Figure 1, *z* = 0 at the surface of the target. We note that in the above model we assumed that the enthalpy of fusion $\Delta H_{m}$ = 0 and the interface laser-target interaction was static. Those assumptions are safe considering the aforementioned laser properties and that only a single pulse are used in this simulation. Moreover, the following initial and boundary conditions are used:

(4) $$T\;{\left(t,z\right)}_{t=0}=T_{0},$$

(5) $$-K_{s}{\frac{\partial T\left(t,z\right)}{\partial z}|}_{z=0}=\frac{\dot{S}}{\mu },\;\;\;\;\;\;\;T\leq T_{m}$$

(6) $$-K_{s}{\frac{\partial T\left(t,z\right)}{\partial z}|}_{z=d}=0,\;\;\;\;\;\;T\leq T_{m}$$

where Equation (4) is the initial condition, $T_{0}$ is the initial temperature, and Equations (5) and (6) are the boundary conditions at the top (z=0) and rear (z=d) surfaces of the YIG target, respectively.

Above the melting temperature, $T>T_{m}$, the target surface will melt and change its physical properties to a liquid phase. Furthermore, if the laser intensity is sufficient, the target surface will reach the boiling point and evaporation will occur. The fluence threshold for YIG can be calculated as follows [24]:

(7) $$F_{th}\approx \frac{\rho_{s}C_{s}\left(T_{m}-T_{0}\right)L_{th}}{\left(1-R\right)},$$

by taking the maximum of the laser penetration depth, $L_{th}=1\;\mu m$, we obtained the fluence threshold $\left(F_{th}\right)$ of YIG ≈ 0.54 J/cm^2^, which is lower than the laser fluence of our system. Thus, we can expect that evaporation will occur during the single-pulsed laser irradiation. The heat conduction on the target for $t>t_{m}$ until the end of the pulse $t=t_{L}$ can be expressed as followed:

(8) $$C_{l}\rho_{l}\frac{\partial T\left(t,z\right)}{\partial t}-C_{l}\rho_{l}V_{r}\frac{\partial T\left(t,z\right)}{\partial z}=\frac{\partial }{\partial z}\;\left(K_{s}\frac{\partial T\left(t,z\right)}{\partial z}\right)+\dot{S},$$

where $C_{l},\rho_{l},\;K_{l},$ are the density, specific heat, and thermal conductivity of the liquid phase of YIG, respectively. Above the boiling point, the recession rate of evaporated material, $V_{r}$, can be described by the Hertz–Knudsen equation [13]:

(9) $$V_{r}\;\approx \frac{P}{\rho_{s}\sqrt{\frac{2\pi k_{B}T_{S}}{m}}}\left(1-\beta \right),$$

where $T_{S}$ is the surface target temperature, m is the average atom mass (here, we assumed *m* = 89 by the mass of the Y atom), β is the fraction of vapor particles that return to the surface, and $k_{B}$ is the Boltzmann constant. Moreover, the pressure (P) of ablated material as a function of surface target temperature can be expressed as [13]:

(10) $$P=P_{b}exp\left\{\frac{\Delta H_{v}m}{k_{B}}\left(\frac{1}{T_{b}}-\frac{1}{T}\right)\right\},$$

where $\Delta H_{v}$ is the enthalpy of vaporization at boiling point $T_{b}$ and atmospheric pressure $P_{b}$ (1 atm.). Furthermore, the boundary conditions are expressed as:

(11) $$-K_{l}{\frac{\partial T\left(z,t\right)}{\partial z}|}_{z=0}=-\Delta H_{v}\rho_{l}V_{r}+\frac{\dot{S}\;}{\mu },$$

(12) $$-K_{l}{\frac{\partial T\left(z,t\right)}{\partial z}|}_{z=d}=0$$

Upon vaporization, the vapor material will also absorb the laser energy, causing its temperature to increase, and ionization will occur, leading to the formation of the plasma plume. The absorption of laser energy by the vapor material is dominated by inverse bremsstrahlung (IB) and the photoionization mechanism (PI) [24]. The IB process involves the absorption of laser energy by a free electron, while the PI happens when the electron is excited directly due to laser energy absorption by neutral atoms. These two mechanisms lead to various species incorporated within the plasma plume, such as electrons, ions, neutrals, clusters, and particulates. 

At the end of the laser pulse, plasma with a particular height *H* will be formed. Due to the density and absorption of the plasma plume, now the laser energy that reaches the surface of the target will be reduced, which is known as the plasma shielding effect [20]. Therefore, the source term can be written as followed:

(13) $$\dot{S}=\mu \left(1-R\right)I\left(t\right)e^{-\mu z}e^{-\left(\alpha_{IB}+\alpha_{PI}\right)H},$$

where $\alpha_{IB}$ and $\alpha_{PI}$ are the absorption coefficients of the IB and PI absorption mechanisms, respectively. The absorption coefficient of IB is given by [25]:

(14) $$\alpha_{IB}=3.69\times 10^{8}\left(\frac{Z_{IB}{}^{3}N_{IB}^{2}}{T_{v}{}^{0.5}\nu^{3}}\right)\left\{1-e^{\left(-\frac{h\nu }{k_{b}T_{v}}\right)}\right\},$$

where $Z_{IB}$, $N_{IB}$, *h*, $T_{v}$, and ν are average charge, ion density, Plank constant, vapor temperature, and frequency of laser, respectively. Moreover, the absorption coefficient of IB is given by [25]:

(15) $$\alpha_{PI}=\sigma_{PI}N_{PI},$$

where $N_{PI}$ is the neutral atom density and $\sigma_{PI}$ is the cross-section between an excited neutral atom and a photon that is involved in the photoionization process. The typical value of $\sigma_{PI}$ is in the range of 10^−21^ m^2^ [26].

### 2.2. Plasma Plume Expansion

After the termination of the laser pulse ($t=t_{L}$), the existing plume will expand away from the target surface due to the conversion of high thermal energy and energy stored as excitation and ionization in the plasma to kinetic energy [10]. In nano-second laser irradiation and with the laser properties under consideration, where the repetition rate is 10 Hz, we can assume the plume expansion time to be far longer than its formation time (close to pulsed duration, ~15 ns). Thus, the plume expansion can be considered independent of its formation.

**Figure 2:**
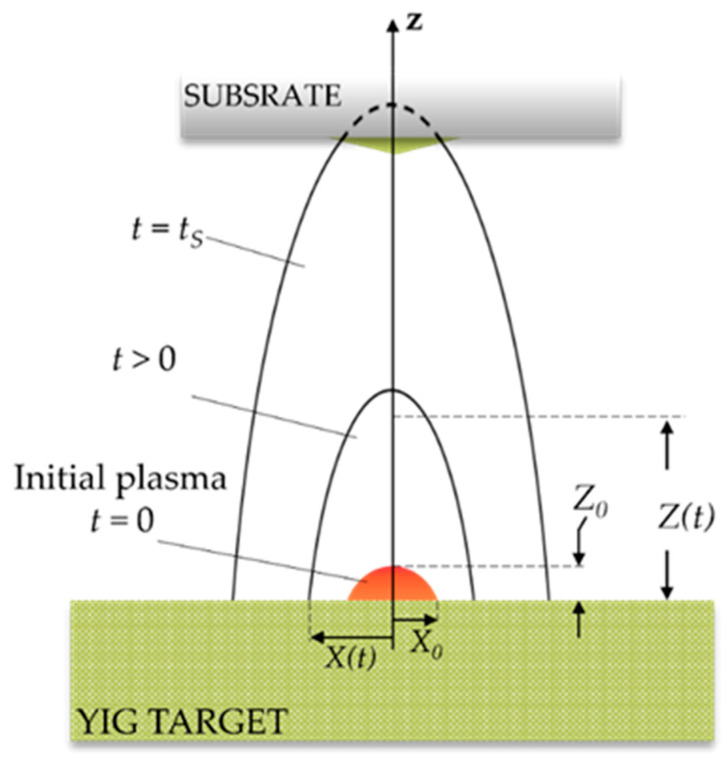
Schematic of the plasma plume expansion after termination of the laser pulse ($t=t_{L}=0$). The initial plasma plume, with dimensions of height ($Z_{0}$) and radius ($X_{0},Y_{0}$) at (t=0), expands (*X*(*t*), *Y*(*t*), *Z*(*t*)) at *t* > 0) as a semi-ellipsoid perpendicular to the target surface (*z*-axis). The time when the edge of the plasma plume reached the substrate is denoted as $t_{S}$.

Figure 2 displays the schematic of the plasma plume expansion after the termination of the laser pulse, showing the change in plume dimension over time. The initial plasma with dimensions of height: $Z_{0}$ and radius: $X_{0}$ and $Y_{0}$ expands as a semi-ellipsoid with a front determined by the axes X(t), Y(t) and Z(t). Here, the radius can be approximated by the laser beam radius, $X_{0}=Y_{0}=$ 4.75 mm. The height of the initial plume is much smaller than the radial dimension ($Z_{0}\ll X_{0},\;Y_{0})$. The height of the plume in *z*-direction can be approximated by $Z_{0}\approx \upsilon_{s}\times t_{L}$ where $\upsilon_{s}$ is the sound velocity in the plume [27]. When the plasma plume is modeled as an ideal gas, the speed velocity can be calculated as [25]:

(16) $$\upsilon_{s}=\sqrt{\frac{\gamma k_{B}T_{P}}{m}},$$

where γ is the specific heat ratio or adiabatic index, $k_{B}$ is the Boltzmann constant, and $T_{P}$ is the initial plasma plume temperature. For a monoatomic ideal gas, γ = 5/3; however, in laser-produced plasma, the value of γ is expected to decrease because of the high plasma temperature and high degree of excitation, as well as the ionization of the plasma species. The value of γ is estimated in the range of 1.2–1.3 [21,28].

In this study, we simulate the plume expansion under vacuum conditions. The plasma plume is modeled as an ideal gas that undergoes adiabatic expansion. The expansion of the plasma plume in Cartesian coordinate can be expressed as [27]:

(17) $$Z\frac{d^{2}Z}{dt^{2}}=X\frac{d^{2}X}{dt^{2}}=X\frac{d^{2}X}{dt^{2}}=\left(5\gamma -3\right)\left(\frac{E}{M}\right){\left(\frac{X_{0}Y_{0}Z_{0}}{XYZ}\right)}^{\gamma -1},$$

where $\gamma =C_{p}/C_{v}$ is the adiabatic constant, and *X*, *Y,* and *Z* are the dimensions of the plume as a function of *t* in the axes of x, y, and z, respectively. *E* is the initial energy and *M* is the mass of the plume, where the ratio can be approximated by EM≈υs2=γkBTPm. The model is based on Lie group transformation theory, where the solution of the gas dynamic equation is simplified. In this model, the density and the pressure of the plume are constant on the ellipsoidal surface, e.g., $\frac{x^{2}}{X{\left(t\right)}^{2}}+$ $\frac{y^{2}}{Y{\left(t\right)}^{2}}+\frac{z^{2}}{Z{\left(t\right)}^{2}}=$ constant. Furthermore, the hydrodynamic motion of all particles in the plume is governed by self-similarity, such that the velocity is controlled by the relative position of the edge [10,23]. Therefore, the velocity distribution in *x*, *y*, and *z*-axes can be calculated as:

(18) $$v_{x}=\frac{x}{X}\frac{dX\left(t\right)}{dt},v_{y}=\frac{y}{Y}\frac{dY\left(t\right)}{dt},v_{z}=\frac{z}{Z}\frac{dZ\left(t\right)}{dt}$$

## 3. Numerical Implementation

### 3.1. Laser Parameters and Material Properties

Table 1 and Table 2 summarize the laser parameters, and the physical properties of YIG target materials.

Several properties of liquid-YIG that cannot be found in references are set to be the same values as in the solid phase. To the best of our knowledge, the enthalpy of vaporization $\Delta H_{v}$ has not been determined experimentally. Therefore, we set the value to be in the order of the corresponding oxides. 

### 3.2. Simulation of the Temperature of the YIG Target

To simulate the effect of laser pulse radiation on the temperature of the YIG target, we solve the 1D heat conduction partial differential equation (Equation (3)) by using the finite different numerical (FDM) method. Two source terms are assigned to each equation, for temperature below melting point $\left(T\leq T_{m}\right)$ and above melting point $\left(T>T_{m}\right)$, expressed in Equation (1) and Equation (13), respectively.

Two different domains are employed, where *n* is for time domain (*t*) and *j* is for the depth from the surface of the target domain (*z*). Based on the known initial condition at *t* = 0, where the initial temperature *T* = 300 K is independent of depth, we can use a center approach for the *z*-domain; meanwhile, the forward approach is applicable for the *t*-domain. The number of partitions for the time and depth domains are assigned to *M* and *N*, respectively. The number of partitions determines the calculation results, where *M* >> *N* led to a convergence value.

Numerical expression for heat conduction equation below melting point $\left(t\leq t_{m}\right)$:

(19) $$T_{n+1,j}=T_{n,j}+\frac{\Delta t}{\;C_{s}\rho_{s}}\left\{\frac{K_{s}}{\Delta z^{2}}\;\left(T_{n,j+1}-2T_{n,j}+T_{n,j-1}\right)+\left(1-R\right)\mu I_{n}\exp\left(-\mu z\right)\right\},$$

with boundary conditions:

(20) $$T_{n+1,1}=T_{n+1,2}+\frac{\Delta z}{K_{s}}\left(1-R\right)I_{n},$$

(21) $$T_{n+1,N}=T_{n+1,N-1,}$$

and initial temperature $T_{1,j}$ = 300 K. Furthermore, for temperatures above the melting point $\left(t_{M}<t<t_{L}\right),$ the numerical equation can be express as:

(22) $$\begin{array}{l} T_{n+1,j}=T_{n,j}+\frac{\Delta t}{\;C_{l}\rho_{l}}\{\frac{K_{s}}{\Delta z^{2}}\;\left(T_{n,j+1}-2T_{n,j}+T_{n,j-1}\right)\\ +\left(C_{l}\rho_{l}V_{r}(T_{n,1}\right)+\left(1-R\right)\mu I_{n}\exp\left(-\mu z\right)\exp\left(-\left(\alpha_{IB}{}_{n}+\alpha_{PI}{}_{n}\right)H\right)\} \end{array}$$

(23) $$Vr\;\approx \frac{P_{b}exp\left\{\frac{\Delta H_{v}\left(T_{b}\right)m}{k_{B}}\left(\frac{1}{T_{b}}-\frac{1}{T_{n,1}}\right)\right\}}{\rho {\left(\frac{2\pi k_{B}T_{n,1}}{m}\right)}^{\frac{1}{2}}}\left(1-\beta \right)$$

with boundary condition:

(24) $$T_{n+1,1}=T_{n+1,2}+\frac{\Delta z}{K_{s}}\left(1-R\right)I_{n}\exp\left(-\mu z\right)\exp(-\left(\alpha_{IB}{}_{n}+\alpha_{PI}{}_{n}\right)H-\Delta H_{v}\rho_{l}V_{r},$$

(25) $$T_{n+1,N}=T_{n+1,N-1},$$

and the initial temperatures for each depth are set to the melting temperature, $t=t_{m}$, $T_{1,j}=T_{m,j}$,. The shielding effect of the plasma plume decreases the laser incident intensity which reaches the surface target. The rate of decrease is determined by the absorption coefficient IB $\left(\alpha_{IB}\right)$ and the PI $\left(\alpha_{PI}\right)$ mechanism, which can be expressed as:

(26) $$\alpha_{IB}\left(n\right)=3.69\times 10^{8}\left(\frac{Z_{IB}{}^{3}N_{IB}^{2}}{T_{v}{{}_{n,1}}^{0.5}\nu^{3}}\right)\left\{1-e^{\left(-\frac{h\nu }{k_{b}T_{v}{}_{n,1}}\right)}\right\},$$

(27) $$\alpha_{PI}\left(n\right)=N_{PI}\sigma_{PI,x},$$

where for such local thermal equilibrium (LTE) of the plume, the average charge $Z_{IB}\approx$ 2 [20], $\upsilon =5.64\times 10^{14}$, and $T_{v}$ is assumed to be the same with the boiling temperature *T_b_*, while $\sigma_{PI,x}$ is set to 10^−21^ m^2^. In this calculation, the density of the ion ($N_{IB})$ and the neutral atom density ($N_{PI})$ are set as a function of time, where the maximum values are reached at the end of the laser pulse $(t_{L}),$ as followed:

(28) $$N_{IB}\left(n\right)=N_{PI}\left(n\right)={\left(n-1\right)}^{10}\left(\frac{N_{max}}{{\left(M-1\right)}^{10}}\right),$$

where $N_{max}$ is set to 10^21^/cm^3^. As shown in Equation (13), the decrease in laser intensity is also a function of plume height (*H*), which is expressed as:

(29) $$H\left(n\right)=\left(n-1\right)\left(\frac{H_{max}}{M-1}\right),$$

where $H_{max}$ is the maximum height of the plume. Just as for $N_{IB}$ and $N_{PI}$, the height of the plume is set to reach the maximum at the end of the laser pulse.

### 3.3. Simulation of Plasma Plume Expansion

The geometry of plasma plume expansion after the termination of the laser pulse is depicted in Figure 2. We calculate dimension and edge velocity for the equation plume expansion (Equation (17)) by using the ordinary differential equation, ode45 function in MATLAB^®^. The initial plume dimension $X_{0}=Y_{0}$ is estimated by a laser beam radius of 4.75 mm, while $Z_{0}$ = $H_{max}$ is estimated from sound velocity (Equation (16)), which is dependent of the initial plasma temperature (*T_P_*). Since the initial plume has much larger thermal energy than the kinetic energy, the initial condition for the edge velocity can be set as:

(30) $$\frac{dX\left(0\right)}{dt}=\frac{dY\left(0\right)}{dt}=\frac{dZ\left(0\right)}{dt}=0,$$

and the calculation is conducted for an expansion time of 10 µm with time step Δt = 0.1 ns. 

As one can see from Equation (17), the values of γ, E, and *M* are critical to obtaining the correct calculation result. However, to the best of our knowledge, those values have not been experimentally confirmed yet. Therefore, we input the estimation values for γ, E, and *M* in our calculation. The ratio of $\frac{E}{M}\approx \nu_{s}{}^{2}$, which depends on the temperature of the initial plasma. We evaluate the expansion by varying the *T_P_*. The range for the initial plasma temperature is set to 7000 K–20,000 K, referring to the previous study of nano-second laser ablation of a YBa_2_Cu_3_O_7_ target [21].

## 4. Result and Discussion

The theoretical models consisting of laser-target heating (Equations (3) and (8)) and plume expansion (Equation (17)) were numerically simulated. The thermophysical properties of YIG used in the calculation are summarized in Table 2. Further, Table 3 summarizes the parameters of the plasma plume used in the calculation:

We choose one value of *γ* = 1.2 and varied the plasma temperatures, resulting in the three different values for height and the ratio of the energy and mass of the initial plasma plume ($t=t_{L}$).

**Figure 3:**
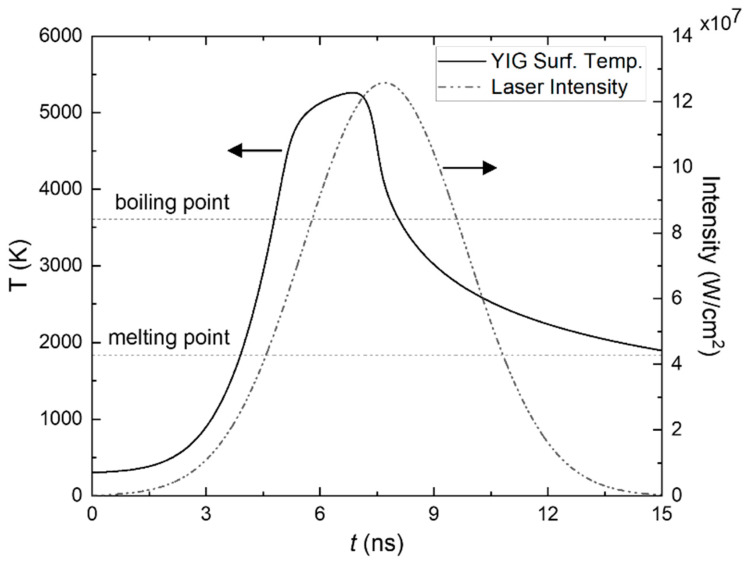
Effect of laser irradiance on the surface temperature of the YIG target. The left *y*-axis denotes the surface temperature (T), while the right *y*-axis denotes the intensity of laser, I(t). T was calculated by numerically solving 1D heat conduction equations as expressed in the Equation (3) for temperatures under the melting point ($T\;\leq \;T_{m}$) and Equation (8) for *T* above the melting point ($T>T_{m}$). *γ* = 1.2 and *T_P_* = 7000 K, resulting in maximum height of plume $H_{max}=$ 13.29 µm, are used in the calculation. The numbers of partitions are M = 12,000 and N = 300.

Figure 3 shows the surface temperature of the YIG target during a single pulse shot, where the left *y*-axis denotes the surface temperature (*T*), while the right *y*-axis denotes the intensity of laser (*I*(*t*)). For this calculation, we employed γ=1.2 and $T_{P}$ = 7000 K, resulting a maximum height of the plume $H_{max}=$ 13.29 µm. The temperature rises because of the absorption of the laser energy by the target and reaches the melting point ($T_{m}$ = 1828 K) at *t* = 3.8 ns. At this region (*t* = 0–3.8 ns), the calculation corresponds to the first 1D heat conduction equation (Equation (3)). After the target surface liquidizes, the surface temperature rises further and reaches the boiling point ($T_{m}$ = 3611 K), when *t* = 4.8 ns. It can be seen that the temperature of target surface increases further, surpassing the boiling point and reaching a maximum of about *T* = 5250 K before decreasing exponentially. It is interesting to see that the surface temperature is saturated even before the laser reach its maximum intensity. This result implies that the shielding effect of the plasma plume reduces the intensity of laser to reach the target surface. In the calculation, we included the term for plasma absorption, which reduced the source heat term used in Equation (8) by rate of $e^{-\left(\alpha_{IB}+\alpha_{PI}\right)H}$. Furthermore, we also considered the liquid-vapor phase change by taking into account the recession rate of evaporation material $v_{r}$ (Equation (9)), which also contributed to the temperature decrease at the surface after evaporation occurred.

The models with the plasma shielding effect in nano-second laser solid target ablation had been previously developed [20,26]. Zhang et al. [20] evaluate the accuracy of the model with the ablation depth in the target experimentally and compare their model to the model with no plasma shielding effect of Singh et al. [21]. The model with the plasma shielding effect gives better accuracy (~3% error) compared to that of Singh et al. (~10% error) [12,20,21]. Therefore, in the above YIG target temperature model, we incorporated the plasma shielding effect, based on IB and the PI mechanism for moderate laser fluence. However, the accuracy of our calculation still needs confirmation from the experiment, which yet remains for further study.

**Figure 4:**
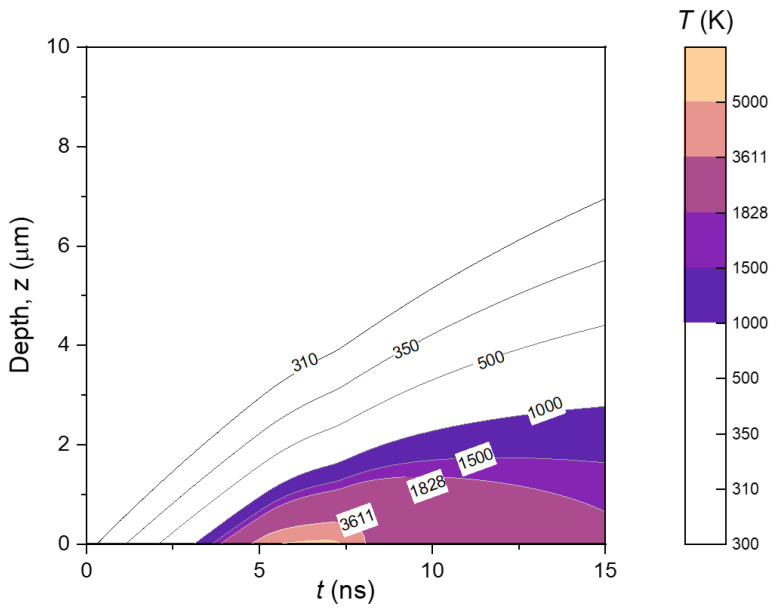
Temperature distribution inside the YIG target during laser pulse irradiation (*t* ≤ 15 ns) calculated for maximum depth *z* = 10 µm. Numerical parameters used as the same as the result shown in Figure 3.

The distribution of temperature inside the YIG target during the laser pulse duration is shown in Figure 4. Temperature distribution inside the target material during laser pulse irradiation (*t* ≤ 15 ns) is calculated for a maximum depth *z* = 10 µm. The range for depth, *z,* is set to 10 µm. The curve inside the graph represents the isothermal boundary line. As expected, the maximum temperature is found at the surface of the target. The melting depth reached ~1 µm from the surface, as shown by the isothermal line for *T >* 1828 K. Even though the surface temperature drops significantly after *t* = 7 ns, a significant region remains molten for the rest of the laser pulse. Furthermore, the evaporation window, when the temperature target surpassed the melting point, is obtained as deep as ~0.4 µm in the 4.8–8 ns radiation period, at which the laser radiation reaches the maximum (t=7.7 ns). This result implies that the supply of evaporated material available to form a plasma is limited to that period. Finally, during laser radiation, the conduction of heat can reach a depth of approximately 7 µm, indicated by the isothermal line of 310 K.

**Figure 5:**
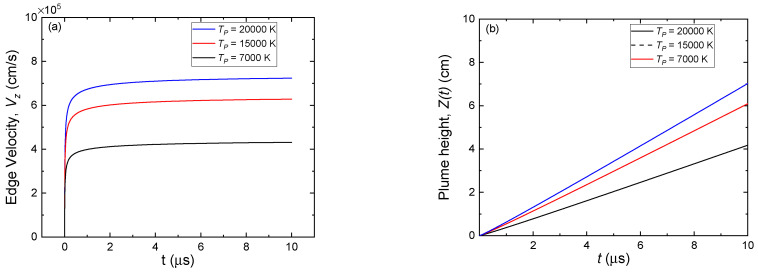
Plume expansion after the termination of laser for initial plasma plume temperature, $T_{P}$ = 20,000, 15,000 and 7000 K. (**a**) Edge velocity or center mass velocity, $V_{z}$ and (**b**) The height of plume edge in the *z*-direction, Z(t).

The calculation of the plume expansion (represented by Equation (17)) for three different initial plume temperatures, *T_P_* = 20,000, 15,000, and 7000 K, is shown in Figure 5. Different initial temperatures of the plasma plume resulted in different initial plume heights ($Z_{0}$), as summarized in Table 1, where a higher plasma temperature led to a higher initial plume height. The edge velocity in the *z*-direction, or the so-called center of mass velocity $(V_{z}$), is shown in Figure 5a. It can be seen that the edge velocity increases rapidly at an early stage of expansion (*t* = 7 ns) before reaching an asymptotic value. The highest velocity is obtained when *T_P_* = 20,000 K, with a maximum of approximately $7\times 10^{5}$ cm/s. Further, Figure 5b shows the evolution of the plume height. Similar dependency on the temperature of the plasma is also observed, where at the end of calculation time of 10 µm, a maximum plume height of 7 cm is obtained for *T_P_* = 20,000 K.

**Figure 6:**
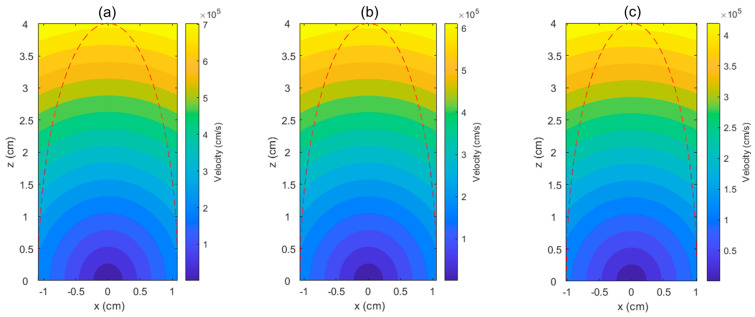
Velocity distribution inside the expanding plasma plume in different initial plasma temperatures, (**a**) *T_P_* = 20,000 K, (**b**) 15,000 K, and (**c**) 7000 K. Red-dashed line represent the semi-ellipsoid plasma plume dimension at height of 4 cm.

Figure 6 shows the velocity distributions inside the plume for each plasma temperature when the plume height is 4 cm. The red dashed line denotes the plume dimensions. The velocity shown here is the resultant velocity $V_{R}=sqrt\;\left(V_{x}{}^{2}+V_{y}{}^{2}+V_{z}{}^{2}\right).$ Self-similar gas dynamics is used to calculate the velocity distribution from the edge velocity, as express in Equation (18). It can be seen that, at a height of 4 cm, the radius of plume only reached approximately 1 cm, which implies that the edge velocity in the *x*, *y*-direction is much smaller than that of z-direction. In fact, for *T_P_* = 20,000 K, the ratio $V_{x}/V_{z}$ is 0.2 when the plume height is 4 cm. This edge velocity difference in the radial and perpendicular direction leads to a semi-ellipsoid shape of expansion of the plume, elongated perpendicularly to the sample surface. Furthermore, the dependence of the plasma temperature clearly shows where a higher plasma temperature led to a higher velocity distribution inside the plume, where for *T_P_* = 20,000, 15,000, and 7000 K, the maximum resultant velocities, *V_R_*, of approximately 7, 6.1, and 4.2 × 10^5^ cm/s are achieved, respectively. 

It was previously demonstrated experimentally, in SrTiO_3_ grown by PLD on a Si substrate, that a high energy of plasma can induce visible burning and destroy the crystallinity of the substrate [33]. Even though, after post-annealing, the Si substrate crystallinity showed improvement, the existence of secondary phases was observed on the grown SrTiO_3_ film. In the study considered here, the plasma plume expansion in vacuum was simulated, as shown in Figure 6, wherein the velocity of the ablated particles was calculated. However, the kinetic energy will be dependent on the ablated species and its respective density, which should be evaluated further by direct measurement, for instance, by using laser-induced breakdown spectroscopy (LIBS) [11]. 

## 5. Summary

In summary, we have studied the effect of nano-laser radiation on the temperature of the YIG target. Within the laser parameters under consideration, a single pulse of laser increases the YIG target’s temperature to above the boiling temperature and generates a plasma. Furthermore, the expansion of the plasma plume in vacuum was also simulated. It shows that the kinetic of the expanding plasma plume strongly depends on the temperature of the initial plasma plume. These results can be used as an input to set the growth parameters of pulsed laser deposition of YIG thin films, such as the laser fluence and the substrate-target distances.

## Figures and Tables

**Table 1:** Laser parameters used for simulation. Referring to parameters of Nd:YAG Continuum Surelite III model.

Parameters	Symbol	Value
Wavelength	λ	532 nm
Laser Energy	E	446 mJ
Repetition Rate	-	10 Hz
Beam Diameter	D	9.5 mm
Pulsed Width (FWHM)	*w*	5 ns
Laser Fluence	ϕ	0.63 J/cm^2^
Peak Intensity	$I_{max}$	1.26 × 10^8^ W/cm^2^

**Table 2:** Thermal and optical properties of Yttrium-Iron Garnet (YIG) used for simulation.

Properties	Symbol	Value	Refs.
Solid phase thermal conductivity	$K_{s}$	7.4 J/smK	[29]
Liquid phase thermal conductivity	$K_{l}$	7.4 J/smK	-
Solid phase density	$\rho_{s}$	5170 kg/m^3^	[29]
Liquid phase density	$\rho_{l}$	5170 kg/m^3^	-
Enthalpy of vaporization	$\Delta H_{v}$	8 × 10^6^ J/kg	-
Reflectivity	*R*	0.13	[30]
Solid phase specific heat	$C_{s}$	590 J/kgK	[29]
Liquid phase specific heat	$C_{l}$	590 J/kgK	-
Thermal diffusivity	α	2.4 × 10^−6^ m^2^/s	calculated
Melting point	$T_{m}$	1828 K	[29]
Boiling point	$T_{b}$	3611 K	[31]
Absorption coefficient (@532 nm)	μ	2.5 × 10^6^ m^−1^	[32]

**Table 3:** Plasma plume parameters used in the calculation.

*γ*	*T_P_* (K)	*Z*_0_ = *H_max_* (µm)	*E*/*M* (J/kg)
1.2	7000	13.29	$7.9\times 10^{5}$
1.2	15,000	19.45	$1.7\times 10^{6}$
1.2	20,000	22.46	$2.2\times 10^{6}$

## Data Availability

Data available upon request from the corresponding author.
